# Disseminated Intravascular Coagulation: A Death Trap?

**DOI:** 10.4269/ajtmh.26-0178

**Published:** 2026-11-06

**Authors:** Sakan Binte Imran

**Affiliations:** Sir Salimullah Medical College, Dhaka, Bangladesh

There were only 28 minutes remaining before the end of my shift. I was dreaming of rushing back to my house after 14 hours of hectic duty. I was getting restless. Being the only evening doctor entrusted with four wards, I had a really tough time managing the patients that day. I was on the second floor checking the vitals of a dengue patient when a group of people running to and fro rushed into the ward.

They screamed, “Doctor! Doctor! Anyone there? Doctor!” I, moving a bit away from the patient’s bed, signaled I was there.

I asked them, “What’s the matter? Why are there so many people here? Anything wrong?” Their faces were covered in fear and uncertainty; some women among the crowd were weeping continuously. I could clearly anticipate that something terrible must have happened.

One of them said, “Doctor, my uncle is dying.”

I, not being able to process what they had just said, asked again for clarity. “What happened?”

“My uncle is dying… he is bleeding out. Please help him,” one of the men replied, his voice shaking.

“Where is he?” I asked. “Which ward?”

They did not know the ward specifications. In their panic, all they could tell me was that he was somewhere on the first floor. I grabbed my blood pressure machine and stethoscope and rushed downstairs. They followed behind me, footsteps echoing through the corridor, their voices filled with desperation.

“Where is he?” I asked again as we reached the floor.

They pointed toward Unit 8, the male ward; the second ward on the right-hand side of the corridor.

The moment I entered, I froze for a second. The scene before me was something I had never witnessed before.

He was bleeding. Not just from one place. From everywhere. Blood stained his clothes, the bed sheets beneath him, the pillow under his head. He suddenly turned to one side and vomited blood right in front of my eyes. Dark red fluid spilled from his mouth while blood continued to seep from his anal orifice. The smell of blood hung heavily in the air.

Until that moment, I had never seen someone bleed from all their orifices. But as people say, there is a first time for everything.

And that was mine.

My mind snapped back to action. I rushed to the nurses’ office with the patient’s file and quickly gave the orders: 1 liter normal saline stat, initial 500 mL to run immediately and the remaining 500 mL at 20 drops per minute, with an injection of tranexamic acid stat. Within seconds, the nurses were preparing the fluids.

I returned to the bedside and began checking his vitals. His pulse was barely palpable. Weak. Thready. When I measured his blood pressure, it was 60/40 mm Hg. His skin felt cold under my fingers.

This was bad. Very bad.

I immediately called my superior. The first call went unanswered. Then the second. My heart was racing almost as fast as the chaos unfolding in the ward. Finally, on the third attempt, he picked up.

I explained everything rapidly in my trembling voice: the bleeding, the vitals, the condition of the patient.

He listened and then calmly instructed me to send urgent investigations: complete blood count, serum fibrinogen, prothrombin time, activated partial thromboplastin time, D-dimer, and blood grouping with crossmatch.

Even before the reports came, a dreadful diagnosis had already begun forming in my mind. The patient had been admitted earlier for exacerbation of chronic obstructive pulmonary disease secondary to bacterial infection with *Haemophilus influenzae*. Just hours ago, he was another patient struggling to breathe. None of us imagined how quickly a condition that started in the lungs could spiral into something as catastrophic as bleeding from multiple sites of the body. Medicine can sometimes change directions within hours, reminding us how fragile the human body truly is.

Blood samples were drawn and rushed to the laboratory.

And then the waiting began. Four hours. Four painfully long hours where every minute felt heavier than the last.

In underprivileged countries like Bangladesh, delayed access to rapid laboratory investigations and critical resources remains a real challenge. If I had an iStat machine that could provide results in a matter of minutes instead of hours, then I would have known what to prioritize—platelets first? Or fibrinogen? And I would not have wasted time and resources giving blood products that might not be the ones most needed. I could have also monitored progress. With a well-stocked blood bank, I would not have worried that giving this patient another three units of a blood product would mean that three other patients would have to go without.

Meanwhile, we did not stop. Empirical supportive treatment continued while we awaited laboratory confirmation. The nurses kept replacing blood-soaked sheets. The family members stood outside the ward whispering prayers, their eyes fixed on the door as if hoping someone would come out and say everything was under control. Inside the ward, however, the battle continued.

When the reports finally came back, they confirmed what we feared. Platelets had dropped drastically. Coagulation parameters were severely deranged. Fibrinogen levels were dangerously low. It was disseminated intravascular coagulation.

There was no time to hesitate. Platelet transfusions were started immediately. Fresh-frozen plasma followed. Cryoprecipitate was arranged to replace fibrinogen. One after another, the transfusions began, each bag carrying a silent hope that the bleeding would finally slow down.

But it didn’t.

The blood kept coming. It slowed but didn’t stop. Hour after hour passed. The ward lights remained harsh and bright while the rest of the hospital slowly fell into the quiet stillness of the night. Monitors continued their rhythmic beeping. Nurses moved swiftly between beds. And I stood there watching the patient, hoping, desperately hoping for a sign that the tide was turning.

It never did.

As the first light of morning began to creep through the windows, his pulse grew weaker and weaker. Eventually, the monitor fell silent.

That morning we lost the patient. I stayed the entire night in the hospital and witnessed every moment.

For us in the hospital, it was the loss of a patient. But outside the ward, a family had just lost their father. A wife had just lost her husband. And the universe had just lost a life.

That night left a lesson engraved deeply in my mind. Disseminated intravascular coagulation is like a ticking time bomb. Once it starts, every second matters. The faster you recognize it and act against it, the greater the chance that the patient may survive.

We tried everything we could: platelet transfusions, fresh-frozen plasma, cryoprecipitate, and every possible supportive measure available to us.

But sometimes medicine is not only about knowledge, skill, or effort. Sometimes it is simply about time.

And time, in that case, was something we did not have enough of.

The case also reminded me that prevention matters. Vaccination against *Haemophilus influenzae* had been planned after recovery from the acute infectious phase, but the patient deteriorated before that could be done.

In underprivileged countries like Bangladesh, delayed access to rapid laboratory investigations and critical resources remains a real challenge. We hope for better facilities, faster diagnostics, and stronger healthcare systems so that we can serve our patients better and give them the fighting chance they deserve.

Perhaps if laboratory investigations could be processed faster. Perhaps if critical tests returned in minutes instead of hours. Perhaps if the blood bank had been well stocked, with reliable quality and availability of blood products.

Maybe that story could have ended differently.

